# Work stress, dietary patterns, and physical activity on metabolic and hepatic biomarkers among healthcare professionals in Ghana

**DOI:** 10.1186/s41043-026-01344-4

**Published:** 2026-05-18

**Authors:** Daniel Ayeltigah, Nancy Gyima Wuabu, Mary Amoako, Moses Ofosu Amoako, Collins Afriyie Appiah

**Affiliations:** 1 Department of Biochemistry and Biotechnology, Faculty of Biosciences, College of Science, Kwame Nkrumah University of Science and Technology, Kumasi, Ghana; 2 The Nutrition Unit, Kibi Government Hospital, Eastern Region, Ghana; 3 Family Medicine Directorate, Komfo Anokye Teaching Hospital, Kumasi, Ghana

**Keywords:** Work stress, Metabolic syndrome, Hepatic biomarkers, Physical activity, Dietary pattern

## Abstract

**Objective:**

This study investigated the association between lifestyle exposures (work stress, dietary patterns, and physical activity) and metabolic and hepatic biomarkers among healthcare professionals in Ghana.

**Methods:**

A cross-sectional survey was conducted among 119 participants at the Kibi Government Hospital. The survey included demographic and lifestyle questionnaires, work stress assessments, anthropometric measurements, and biochemical analyses. Metabolic syndrome (MetS) was defined according to the International Diabetes Federation criteria. Binary logistic regression models were applied to determine the associations.

**Results:**

The prevalence of MetS was 11.0%, with high blood pressure (29.7%) and abdominal obesity (57.1%) being the most prevalent MetS components. The majority of participants reported high stress levels (60.4%), and 59.7% engaged in moderate physical activity. Approximately 65.3% had AST/ALT greater than 1. Four dietary patterns were identified: Western Pattern, Traditional mixed pattern, Protein-rich pattern, and Fruit and Dairy pattern. Moderate and high adherence to the Western dietary pattern were significantly associated with increased odds of abdominal obesity (Tertile 2: OR 3.244, 95% CI 1.219–8.629, p-value 0.018, Tertile 3: OR 4.231, 95% CI 1.550-11.546, p-value 0.005). A non-significant association was also observed between elevated triglycerides and ALT (OR 1.03, 95% CI 0.998–1.05, *p* = 0.066).

**Conclusion:**

This study highlights a considerable burden of metabolic risk factors among hospital staff in Ghana, with abdominal obesity and elevated blood pressure being the most prevalent components of metabolic syndrome. High work stress levels, moderate physical activity, and elevated AST/ALT ratios (indicating a predominance of hepatocellular injury and suggesting potential liver dysfunction) were also common. Greater adherence to the western dietary pattern was significantly associated with increased odds of abdominal obesity while marginal association was observed between elevated triglycerides and ALT. These findings highlight the need for regular screening for metabolic risk factors and liver function among health professionals. Healthcare institutions should introduce targeted interventions such as nutrition education and improved access to healthy food options to prevent related complications such as liver cirrhosis and diabetes.

**Supplementary Information:**

The online version contains supplementary material available at 10.1186/s41043-026-01344-4.

## Introduction

Healthcare workers are an important group of professionals whose work is vital to promote a healthy society [1]. Due to their access to medical knowledge and close involvement in healthcare delivery, it is often assumed that the prevalence of metabolic diseases and their modifiable risk factors would be relatively low among healthcare workers [1, 2]. However, the demands of hospital work, including shift schedules, long hours, and high physical and emotional stress, expose health workers to elevated risks for chronic diseases [3].

In sub-Saharan Africa, research shows that healthcare professionals (HCPs) face a rising burden of cardiometabolic risk. In Nigeria, studies report a high prevalence of hypertension and obesity among hospital employees [2], while in Kenya, more than 40% of healthcare staff were found to be obese, predisposing them to an increased risk of metabolic syndrome (MetS) risk [4]. In Ghana, although limited, available research indicates high levels of abdominal obesity, hypertension, and dyslipidemia among health workers [5]. These patterns suggest that HCPs cannot always be considered healthier than the patients they serve [6].

Unhealthy lifestyle practices contribute to this burden. HCPs often consume convenience foods such as fried snacks, baked goods, and sugar-sweetened beverages several times per week, because they are readily accessible during busy work shifts [7]. Skipping meals is also common due to busy work schedule, with many HCPs missing breakfast, lunch, or supper [7]. These behaviours, combined with occupational stress and reduced opportunities for exercise, increase susceptibility to metabolic and hepatic disorders [8]. Over time, these lifestyle changes promote dyslipidemia, chronic inflammation and increased visceral fat accumulation [9]. In Sub-Saharan Africa, rapid urbanization, changing dietary habits, and increasing work-related stress are driving an upsurge in lifestyle-related non-communicable diseases (NCDs) [10].

Hepatic biomarkers play a central role in glucose and lipid metabolism. Abnormal liver enzymes are associated with insulin resistance and other components of MetS [11]. Early changes in these biomarkers such as AST and ALT may indicate sub clinical metabolic dysfunction before symptoms develop [12]. In research, these biomarkers provide measurable outcomes for evaluating the effectiveness of dietary, pharmacological, or lifestyle interventions [13]. However, no prior study in Ghana has examined combined associations between lifestyle factors and hepatic biomarkers among HCPs.

This study, therefore, examined associations between lifestyle exposures, including stress, physical activity, and dietary patterns, onhepatic, nutritional, and metabolic biomarkers among hospital staff at a primary care hospital in Ghana. By identifying specific risk factors, these findings provide evidence to inform targeted interventions aimed at reducing the burden of MetS and other chronic conditions among HCPs, a key workforce critical to achieving universal health coverage. Furthermore, this study contributes to global discussions on workplace wellness and supports the achievements of the United Nations Sustainable Development Goals, particularly SDG 3 and SDG 12, which seek to improve health and well-being among individuals and promote responsible consumption. In this research, we hypothesized that higher levels of work stress, poor dietary patterns, and low physical activity were associated with abnormal liver enzymes and MetS among healthcare professionals.

## Methodology

### Study design and setting

This study employed a cross-sectional study design and a convenience sampling method to collect data from 119 health workers at the Kibi Government Hospital, a primary care hospital situated in the Abuakwa South Municipality of the Eastern Region of Ghana.

### Sample and study population

The sample size was determined using Cochran’s formula, based on a conservative prevalence of 6% for metabolic syndrome [14] a 95% confidence level, and 5% margin of error. Although our primary outcomes are metabolic and hepatic markers, the prevalence of these markers could be higher or lower than that of metabolic syndrome. Using a prevalence of 6% therefore ensures that the study is adequately powered to detect meaningful differences in the intended outcomes.

Cochran’s Formula; $$\begin{gathered}{n_{0}=\frac{Z^{2}\cdot{p}\cdot(1-p)}{e^{2}}}\end{gathered}$$  

$$\begin{gathered}{n_{0}=\frac{(1.96)^{2}\cdot{0.06}\cdot(1-0.06)}{(0.02)^{2}}}\end{gathered}$$  

n_0_ = 86.49_

n_0_ = 87 participants.

Although the calculation indicated a required sample size of 87 participants, a total of 119 participants were recruited to enhance precision and representativeness. Recruitment was conducted through the hospital’s internal communication channels. Eligible participants were full-time staff aged 20–50 years, who were not pregnant, had no diagnosed chronic condition, and had a minimum of six months of work experience. The study sample consisted of HCPs from diverse wards, departments, and units, including doctors, pharmacists, nurses, nutritionists, laboratory technicians, and orderlies.

### Data collection

Questionnaires were administered in person by trained data collectors. A structured questionnaire was used to obtain information on participants’ sociodemographic characteristics, lifestyle factors (dietary patterns, physical activity, alcohol intake), and work-related stress. Data on lipid profiles [total cholesterol, low-density lipoprotein (LDL), high-density lipoprotein (HDL), triglycerides and liver enzymes [Alanine aminotransferase (ALT), aspartate aminotransferase (AST), Gamma-glutamyl Transferase (GGT)] were retrieved from the Laboratory Information Management System (LIMS, Kibi Government Hospital, Ghana). These data were collected six months before the questionnaire administration as part of the hospital’s annual staff screening. This was considered satisfactory because generally eating patterns and physical activity are usually stable in most populations. However, participants who reported changes in their diet or physical activity during this period were excluded from the study to ensure that the retrospective data accurately reflected stable exposures relevant to the study (stress, diet and physical activity).

### Work stress, physical activity and dietary patterns

The Effort-Reward Imbalance (ERI) model was applied to measure work-related stress by assessing the balance between the effort invested by healthcare workers and the rewards they receive [15]. The ERI evaluates three key dimensions: effort, reward, and over-commitment. Physical activity was measured using the International Physical Activity Questionnaire (IPAQ) [16]. The IPAQ measures the frequency and duration physical activity as well as sedentary behavior. Dietary intake was assessed using a 7-day Food Frequency Questionnaire (FFQ), which comprised 60 food items [17].

### Anthropometric measurement and blood pressure

Waist circumference was measured using a tape measure (WAWAK, United States), following standardized protocols [18]. Blood pressure was assessed using an Omron digital monitor (HEM-7146T2-EBK, Vietnam). Participants were asked to rest for five minutes prior to measurement. Blood pressure was recorded three times, and the average of the readings was used for analysis. Measurements were conducted with the arm positioned at heart level, using an appropriately sized cuff, with participants having avoided caffeine intake or physical exercise in the 30 min prior.

### Definition of MetS

MetS was defined based on the criteria of the International Federation of Diabetes, where abdominal obesity is a compulsory component plus two additional criteria.

1. Waist circumference (cm): Males ≥ 94 and Females ≥ 80.
2. HDL cholesterol (mmol/L): Males < 1.04 and Females < 1.30.
3. Triglycerides (mmol/L): ≥ 1.70.
4. Fasting glucose (mmol/L): ≥ 5.6.
5. Blood pressure (mmHg): ≥ 130/85.

### Data analysis

The collected data were entered into SPSS version 27 for analysis. Chi-square tests were used to determine the differences between clinical and non-clinical staff, while independent t-tests were applied to compare means ± standard deviations. The 60 food items were divided into 14 groups and used for principal component analysis (PCA) to identify dietary patterns. Varimax rotation was used in the PCA. The factor loadings were grouped into tertiles to represent the level of consumption of the identified food patterns. Food items with factor loadings ≥ 0.50 were considered to contribute significantly to the dietary patterns. Physical activity was calculated as minutes/days/MET, and the scores were categorised based on the IPAQ scores (low, moderate, and high). Data on work stress were analysed using the formula ERI = effort/ (reward × correction factor), where ratios less than 1 were classified as low stress and values greater than 1 as high stress [15]. For liver function, metabolic risk was assessed using the following indicators: AST/ALT ratio (De Ratio) [19], high GGT (> 37), Low albumin (< 48), high AST (> 37), high ALT (> 20) (Based on the established reference from the health facility. The influence of lifestyle factors and liver enzymes on MetS was examined using binary and multiple binary regression models (see Supplementary Table 3). Binary logistic regression analysis was also done to check the associations between dietary tertiles, liver enzymes, and both MetS and its individual components while the multiple binary logistic regression was used to check the association between the hepatic biomarkers, stress, physical activity and dietary patterns. Given the exploratory nature of the study and the modest sample size, multivariable adjustment for confounding factors (age, sex, and some lifestyle factors) was not performed. Statistical significance was set at *p* < 0.05 for all tests.

## Results

A total of one hundred and nineteen (119) health workers participated in the study. Majority of the clinical staff (81.7%) were females, and 45.1% were married. The majority of the clinical staff (94.4%) had attained tertiary education, compared to 62.5% of non-clinical staff. Similarly, income levels among clinical staff were higher, with 25.4% earning more than 4 000 cedis (350$) per month. In general, clinical staff worked more hours than the non-clinical staff, ranging from less than 6 h (2.8%) to 6–9 h (73.2%). Statistically significant associations were observed for gender (*p* = 0.005), educational level (p **<** 0.001), and monthly income (*p* = 0.007) (Supplementary Table 1).

Table 1 shows the results for physical activity and stress among participants. Among clinical staff, low physical activity was more prevalent (31.0%) as compared to non-clinical staff (22.9%), while high physical activity was less prevalent (8.5%) as compared to non-clinical staff (18.8%). The difference in physical activity observed between the two groups was not statistically significant. By contrast, work stress was significantly higher among clinical staff, with 70.8% reporting high stress compared to 43.9% of non-clinical staff (*p* = 0.006). Additionally, the mean work stress score was greater among clinical staff (0.71 ± 0.46) than non-clinical staff (0.43 ± 0.50), resulting in an overall mean of 0.60 ± 0.48.

**Table 1:** Assessment of physical activity and work stress among the hospital staff

Variable	Category	Clinical Staff *N* (%)	Non-clinical Staff *N* (%)	Total	*P*-value
Physical Activity	Low	22 (31.0)	11 (22.9)	33 (27.7)	0.212
	Moderate	43 (60.6)	28 (58.3)	71 (59.7)	
	High	6 (8.5)	9 (18.8)	15 (12.6)	
	Mean (SD)	1.78 ± 0.59	1.96 ± 0.65	1.85 ± 0.62	0.113
Work stress	Low	19 (29.2)	23 (56.1)	42 (39.6)	0.006*
	High	46 (70.8)	18 (43.9)	64 (60.4)	
	Mean (SD)	0.71 ± 0.46	0.43 ± 0.50	0.60 ± 0.48	0.007*

Work stress data were missing for 13 participants; percentages and means are based on available data. Missing data were recorded as 9999

Table 2 presents the consumption levels of the identified dietary patterns among staff (see Supplementary Table 2). Across all the dietary patterns, clinical staff demonstrated higher levels of consumption compared to non-clinical staff: Western pattern (35.4% vs. 30.0%), Traditional mixed pattern (36.9% vs. 27.5%), Protein-Rich pattern (36.9% vs. 27.5%) and Fruit and Dairy pattern (33.8% vs. 32.5%). In contrast, non-clinical staff had lower consumption levels across all dietary patterns, except for the Fruit and Dairy pattern. Specifically, the proportions were as follows: Western pattern (30.8% vs. 37.5%), Traditional mixed pattern (26.2% vs. 45.0%), and Protein-Rich pattern (27.7% vs. 42.5%). For the Fruit and Dairy pattern, clinical staff (33.8%) had a slightly higher proportion of low consumption compared to non-clinical staff (32.5%).

**Table 2:** Consumption levels of dietary patterns among the hospital staff

Food Patterns	Level	Clinical Staff *N* (%)	Non-clinical Staff *N* (%)	*P*-value
Western Pattern	low	20 (30.8%)	15 (37.5%)	0.465
	moderate	22 (33.8%)	13 (32.5%)	
	high	23 (35.4%)	12 (30.0%)	
Traditional Mixed Pattern	low	17 (26.2%)	18 (45.0%)	0.086
	moderate	24 (36.9%)	11 (27.5%)	
	high	24 (36.9%)	11 (27.5%)	
Protein-Rich Pattern	low	18 (27.7%)	17 (42.5%)	0.142
	moderate	23 (35.4%)	12 (30.0%)	
	high	24 (36.9%)	11 (27.5%)	
Fruit and Dairy Pattern	low	22 (33.8%)	13 (32.5%)	1.000
	moderate	21 (32.3%)	14 (35.0%)	
	high	22 (33.8%)	13 (32.5%)	

The prevalence of MetS and its individual components, and hepatic biomarkers among staff are shown in Table 3. MetS prevalence was higher among non-clinical staff (12.5%) compared with clinical staff (10.0%). Among the components of MetS, abdominal obesity was the most common (57.1%), affecting the majority of clinical staff (60.6%), while high blood pressure (29.7%) was more prevalent among non-clinical staff (41.7%). A high proportion of participants (65.3%) had an AST/ALT ratio greater than 1.

**Table 3:** Prevalence of hepatic biomarkers, MetS and its components

Variable	Clinical Staff *N* (%)	Non-clinical Staff *N* (%)	Total *N* (%)	*P*-value
MetS	7 (10.0%)	6 (12.5%)	13 (11.0%)	0.670
Abdominal obesity	43 (60.6%)	25 (52.1%)	68 (57.1%)	0.359
Elevated FBS	9 (12.7%)	4 (8.3%)	13 (10.9%)	0.456
High blood pressure	15 (21.4%)	20 (41.7%)	35 (29.7%)	0.018
High triglycerides	6 (8.5%)	3 (6.3%)	9 (7.6%)	0.656
Low HDL	20 (28.2%)	9 (18.8%)	29 (24.4%)	0.240
AST/ALT ratio (> 1)	46 (65.7%)	31 (64.6%)	77 (65.3%)	0.899
High GGT	9 (12.7%)	4 (8.3)	13 (10.9%)	0.456
Low albumin	0 (0%)	0 (0%)	0 (0%)	-
High AST	2 (2.8%)	5 (10.6%)	7 (5.9%)	0.078
High ALT	2 (2.9%)	7 (14.6%)	9 (7.6%)	0.018

High blood pressure: missing data = 1, AST/ALT > 1: missing data = 1

AST: missing data = 1, ALT: missing = 1. Analysis was based on the available data. Missing data were recorded as 9999

Table 4 summarizes the binary associations between dietary patterns, work stress, and liver enzymes in relation to MetS. None of these associations were statistically significant (p-value > 0.05).

**Table 4:** Associations between work stress, physical activity, dietary pattern, and liver enzymes on MetS

Variable	OR	95% CI	*P*-value
ERI	1.108	0.245–5.017	0.894
Physical Activity	1.000	0.999-1.000	0.327
Western Pattern	0.726	0.387–1.361	0.318
Traditional Mixed Pattern	0.751	0.392–1.439	0.388
Protein-Rich Pattern	1.316	0.700-2.472	0.394
Fruit and Dairy Pattern	1.508	0.813–2.797	0.193
AST	0.996	0.967–1.027	0.817
ALT	1.020	0.994–1.047	0.133
GGT	1.002	0.971–1.033	0.919
Albumin	0.881	0.678–1.145	0.344

Table 5 presents the associations between work stress, liver enzymes, and components of MetS. The association between ALT and elevated triglycerides was not statistically significant (OR 1.026, 95% CI 0.998–1.054).

**Table 5:** Association between work stress, and liver enzymes on MetS components

Variable	ERI	AST	ALT	GGT	Albumin
FBS	1.122 (0.249–5.063) 0.881	0.993 (0.958–1.030) 0.710	1.016 (0.989–1.044) 0.236	0.963 (0.916–1.012) 0.138	1.023 (0.796–1.314) 0.860
Abdominal obesity	1.672 (0.588–4.756) 0.335	0.981 (0.955–1.008) 0.162	1.003 (0.980–1.025) 0.826	0.999 (0.979–1.019) 0.21	1.082 (0.919–1.274) 0.343
High blood pressure	0.382 (0.108–1.350) 0.135	0.998 (0.980–1.016) 0.845	1.012 (0.990–1.036) 0.291	0.999 (0.977–1.021) 0.939	1.131 (0.944–1.356) 0.183
Low HDL	1.831 (0.576–5.824) 0.305	0.992 (0.965–1.019) 0.540	0.993 (0.964–1.022) 0.617	1.009 (0.987–1.031) 0.414	0.956 (0.792–1.154) 0.638
High triglycerides	1.471 (0.267–8.091) 0.657	0.997 (0.964–1.032) 0.884	1.026 (0.998–1.054) 0.066*	0.983 (0.936–1.032) 0.493	0.887 (0.658–1.195) 0.430

Table 6 summarizes the binary associations between dietary patterns, MetS, its components, and liver enzymes. Participants in the highest tertile of the Western dietary pattern had significantly higher odds of abdominal obesity. (Tertile 2: OR 3.244, 95% CI 1.219–8.629, p-value 0.018,Tertile 3: OR 4.231, 95% CI 1.550-11.546, p-value 0.005). All other associations were not statistically significant (*p* > 0.05).

**Table 6:** Association between dietary patterns on MetS and its components and liver enzymes

Variable	Western Pattern		Traditional Mixed Pattern		Protein-Rich Pattern		Fruit and Dairy Pattern	
	Tertile 2	Tertile 3	Tertile 2	Tertile 3	Tertile 2	Tertile 3	Tertile 2	Tertile 3
MetS	0.293 (0.055–1.566) 0.151	0.644 (0.165–2.522) 0.528	0.806 (0.221–2.932) 0.743	0.146 (0.017–1.289) 0.083*	1.722 (0.378–7.849) 0.482	1.333 (0.275–7.849) 0.721	0.703 (0.145–3.406) 0.662	1.250 (0.306–5.114) 0.756
FBS	0.293 (0.055–1.566) 0.151	0.453 (0.104–1.979) 0.293	1.292 (0.316–5.277) 0.722	0.470 (0.080–2.749) 0.402	1.000 (0.229–4.361) 1.000	0.727 (0.150–3.515) 0.691	1.778 (0.391–8.092) 0.457	1.000 (0.188–5.332) 1.000
High blood pressure	0.856 (0.287–2.556) 0.781	1.788 (0.641–4.990) 0.267	1.156 (0.402–3.318) 0.788	1.382 (0.486–3.926) 0.544	0.962 (0.328–2.817) 0.943	1.449 (0.516–4.073) 0.482	1.300 (0.442–3.827) 0.634	1.696 (0.590–4.875) 0.327
Abdominal obesity	3.244 (1.219–8.629) 0.018*	4.231 (1.550-11.546) 0.005*	0.611(0.230–1.626) 0.324	0.433 (0.0.163–1.146) 0.092	0.702 (0.270–1.824) 0.467	0.788 (0.302–2.054) 0.788	0.706 (0.274–2.955) 0.471	1.128 (0.431–2.955) 0.806
High triglycerides	0.364 (0.066–2.016) 0.247	0.176 (0.020–1.597) 0.123	2.750 (0.496–15.246) 0.247	0.485 (0.042–5.611) 0.563	1.000 (0.188–5.332) 1.000	0.646 (0.101–4.128) 0.645	0.646 (0.101–4.128) 0.645	1.000 (0.188–5.332) 1.000
Low HDL	0.451 (0.145–1.401) 0.169	0.873 (0.314–2.428) 0.794	0.663 (0.237–1.859) 0.435	0.397 (0.129–1.218) 0.106	1.833 (0.614–5.471) 0.277	1.385 (0.451–4.255) 0.570	0.598 (0.187–1.908) 0.385	1.507 (0.538–4.224) 0.435
AST	1.000 (0.060-16.648) 1.000	2.125 (0.184–24.589) 0.546	0.304 (0.030–3.077) 0.313	0.000 (0.000–) 0.998	1.030 (0.062–17.163) 0.983	2.061 (0.178–23.826) 0.563	1.030 (0.062–17.163) 0.983	2.061 (0.178–23.826) 0.563)
ALT/AST > 1	1.000 (0.364–2.744) 1.000	0.688 (0.257–1.838) 0.455	0.776 (0.288–2.087) 0.615	0.878 (0.324–2.384) 0.799	1.278 (0.483–3.377) 0.621	1.667 (0.615–4.519) 0.316	0.462 (0.168–1.269) 0.435	0.663 (0.237–1.859) 0.435
ALT	0.646 (0.101–4.128) 0.645	0.667 (0.104–4.261) 0.668	1.032 (0.193–5.510) 0.970	0.314 (0.031–3.174) 0.326	0.470 (0.080–2.749) 0.402	0.235 (0.025–2.218) 0.206	0.485 (0.042–5.611) 0.563	2.200 (0.371–12.889) 0.382
GGT	1.292 (0.316–5.277) 0.722	0.727 (0.150–3.515) 0.691	2.667 (0.629–11.306) 0.183	0.646 (0.101–4.128) 0.645	3.414 (0.639–18.248) 0.151	2.129 (0.364–12.459) 0.402	1.778 (0.391–8.092) 0.457	1.376 (0.285–6.658) 0.691

## Discussion

In this study, we examined lifestyle exposures and their associations with metabolic and hepatic biomarkers among HCPs in Ghana. Clinical staff reported higher stress levels than non-clinical staff, whereas physical activity was mostly moderate across both groups. No significant associations were found between the lifestyle factors, liver enzymes, and MetS. However, the western food pattern was significantly associated with abdominal obesity. A marginal relationship between ALT and elevated triglycerides, as well as between MetS and Traditional mix pattern, was observed.

### Work stress and physical activity

Clinical staff reported higher levels of work stress compared to non-clinical staff, consistent with our observations. Non-clinical staff also experienced stress, though at lower levels, likely due to organizational pressures such as job insecurity and limited career advancement opportunities [20–22]. Similar findings have been reported in other African settings, showing that clinical roles such as physicians and nurses are exposed to greater psychosocial strain due to high patient loads, night shifts, and emotional demands [21]. Physical activity patterns differed between the two groups. Clinical staff were more likely to engage in moderate activity through walking and standing during shifts, whereas non-clinical staff may have higher activity levels through leisure-time exercise. Both groups, however, include individuals with sedentary work roles, particularly those with desk-based tasks [23–25]. These results reflect variation in activity levels among healthcare staff and show that occupational roles influence daily physical activity, consistent with prior studies in hospital settings [25–26].

### Dietary patterns and consumption levels

Differences in dietary consumption were observed between clinical and non-clinical staff. Clinical staff generally reported higher intake across the identified dietary patterns, likely reflecting greater nutrition knowledge and health awareness. Non-clinical staff were more likely to fall into lower-consumption categories, potentially due to socioeconomic constraints and differences in nutritional knowledge [27–29]. Within clinical staff, fruit and dairy consumption showed wide variability. Some staff incorporated these foods regularly, while others had lower intake, suggesting heterogeneous eating behaviors influenced by lifestyle and practical constraints [30]. Both groups face a shared barrier in the hospital food environment, dominated by affordable, energy-dense meals from vendors and canteens [31–32]. These patterns are consistent with previous studies in Ghana, showing that healthcare professionals often consume Western-style foods like fried snacks and sugar-sweetened beverages due to convenience and accessibility [7].

### Hepatic biomarkers, metabolic syndrome, and its components

The overall prevalence of MetS was relatively low 11% which is lower than 14.8% found among sedentary workers in Ghana [33]. However, individual components were common. Abdominal obesity was observed in more than half of participants, and elevated blood pressure was present in nearly a third of participants. These high rates may reflect occupational and lifestyle factors, including the high levels of work stress reported among clinical staff and frequent consumption of energy-dense foods associated with the Western dietary pattern. This aligns with prior evidence indicating Westernized dietary patterns and occupational stress contribute to individual metabolic risk factors [34–35]. Despite these individual risk factors, many participants did not meet the full criteria for MetS, which requires the presence of at least three components. Additional factors such as the absence of prior chronic disease diagnoses among participants, regular annual health screenings, and the health worker effect may partly explain the relatively low overall prevalence of MetS. Elevated AST/ALT ratios were observed in approximately 65.3% of participants, suggesting early liver dysfunction may be present even in the absence of MetS. Together, these results indicate that although MetS prevalence is relatively low, cardiometabolic risk and early liver dysfunction are present in this workforce.

### Associations between lifestyle factors, liver enzymes, mets, and its individual components

No statistically significant associations were found between overall lifestyle factors work stress, dietary patterns, physical activity and liver enzyme levels or the presence of MetS. This may be due to the cross-sectional design, modest sample size, or relatively short duration of exposure to lifestyle risk factors [36–38]. Several notable trends were observed. ALT showed a marginal association with elevated triglycerides, suggesting subtle hepatic dysfunction may precede overt lipid abnormalities, consistent with prior studies in Asia and the United States [39–40]. Greater adherence to the Western dietary pattern was significantly associated with abdominal obesity, with participants in tertiles 2 and 3 having more than threefold and fourfold higher odds of central adiposity, respectively. Similar associations between Westernized dietary patterns and increased abdominal obesity have been reported in multiple adult populations [41–42]. A marginal trend was observed between the traditional mixed dietary pattern and MetS, indicating a possible protective effect of diets rich in vegetables, cereals, and grains. This aligns with prior evidence that fiber-rich foods, vegetables, and whole grains improve glycemic control, support lipid regulation, and reduce blood pressure [43–45].

### Implications for clinicians and policymakers

The high prevalence of MetS components, stress and elevated liver enzymes reflects the demands of long shifts and poor dietary habits seen among the health workers. These patterns provide the evidence base for clinicians to prioritize early screening for hepatic and metabolic risk factors, even in the absence of disease. Furthermore, the findings suggest that healthcare policymakers should promote workplace wellness programs that address stress, promote healthier food options, and increase physical activity. This will help improve the well-being of health workers in Ghana.

### Strengths and limitations of the study

The strength of this study is its focus on health care professionals. A group that plays an important role in shaping community health behaviours, but is often overlooked in lifestyle and metabolic research in Ghana. The inclusion of both clinical and non-clinical staff allowed for meaningful comparisons across occupational categories, providing insight into how work roles influence lifestyle. Utilizing both lifestyle factors and biological markers also strengthens the robustness of the results. However, the cross-sectional design limits causal interpretations between lifestyle exposures and metabolic or hepatic outcomes. Self-reported data on lifestyle factors may have introduced bias. In addition, there were missing data for some hepatic biomarkers and lifestyle exposures, which could have limited the interpretation of results. A key assumption of this study is that participants’ dietary patterns, physical activity levels, stress exposures, and biomarkers remained relatively stable during the six-month gap between staff health screening and the period of questionnaire administration. While this allowed us to integrate existing laboratory data, we acknowledge the possibility of behavioral or metabolic changes within this period, which could influence observed associations. This study did not adjust for potential confounding variables such as age, sex, lifestyle factors or medication use. As a result, the observed associations should be interpreted with caution. Future studies with larger sample sizes and multivariable analyses are needed to account for potential confounding effects.

## Conclusion

This study highlights that healthcare professionals, despite their role as healthcare providers, are vulnerable to emerging lifestyle-related health risks, particularly abdominal obesity, elevated blood pressure, and early hepatic changes. Although overall associations between lifestyle factors and metabolic syndrome were not significant, strong associations were observed between intake of western dietary patterns and abdominal obesity, with a significant association between elevated triglycerides and ALT levels. These findings highlight the urgent need for workplace wellness programs, healthy food policies and stress-reduction intervention to promote a healthier workforce.

## Electronic Supplementary Material

Supplementary Material 1

## Data Availability

The datasets analyzed during the current study are available from the corresponding author upon reasonable request and with permission from Kibi Government Hospital.
