# Supplementary material for: Work stress, dietary patterns, and physical activity on metabolic and hepatic biomarkers among healthcare professionals in Ghana
Source: J Health Popul Nutr. 2026 May 18;45:173. doi: 10.1186/s41043-026-01344-4 (PMC13386725; doi:10.1186/s41043-026-01344-4)
Supplement: Supplementary file 1 — Supplementary Material 1 [file 41043_2026_1344_MOESM1_ESM.docx]

Supplementary Table 1. Sociodemographic characteristics of participants

| **Variable** | **Category** | **Clinical Staff**  **N (%)** | **Non-clinical Staff N (%)** | **P-value** |
| --- | --- | --- | --- | --- |
| Age | 20-29 | 11 (15.5%) | 13 (27.1%) | 0.095 |
|  | 30-39 | 48 (67.6%) | 23 (47.9%) |  |
|  | 40-50 | 12 (16.9%) | 12 (25.0%) |  |
| Gender | Male | 13 (18.3%) | 20 (41.7%) | **0.005** |
|  | Female | 58 (81.7%) | 28 (58.3%) |  |
| Educational level | No Formal Education | 0 (0.0%) | 0 (0.0%) | **< 0.001** |
|  | Primary | 0 (0.0%) | 1 (2.1%) |  |
|  | JHS | 0 (0.0%) | 8 (16.7%) |  |
|  | SHS | 4 (5.6%) | 9 (18.8%) |  |
|  | Tertiary | 67 (94.4%) | 30 (62.5%) |  |
| Marital status | Single | 37 (52.1%) | 21 (43.8%) | 0.533 |
|  | Married | 32 (45.1%) | 24 (50.0%) |  |
|  | Widow/ Widower | 0 (0.0%) | 0 (0.0%) |  |
|  | Separated/ Divorced | 2 (2.8%) | 2 (4.2%) |  |
|  | Cohabiting | 0 (0.0%) | 1 (2.1%) |  |
| Monthly income | Less 1000 cedis | 3 (4.2%) | 7 (14.6%) | **0.007** |
|  | 1000 to 2900 cedis | 17 (23.9%) | 21 (43.8%) |  |
|  | 3000 to 3900 cedis | 33 (46.5%) | 11 (22.9%) |  |
|  | More than 4000 cedis | 18 (25.4%) | 9 (18.8%) |  |
| Ethnic group | Akan | 46 (64.8%) | 24 (50.0%) | 0.136 |
|  | Ewe | 12 (16.9%) | 7 (14.6%) |  |
|  | Ga Adangbe | 8 (11.3%) | 6 (12.5%) |  |
|  | Grushi | 0 (0.0%) | 1 (2.1%) |  |
|  | Others | 5 (7.0%) | 10 (20.8%) |  |
| Years of experience | 6 months to 1 year | 0 (0.0%) | 1 (2.1%) | 0.591 |
|  | 2 years to 5 years | 36 (50.7%) | 21 (43.8%) |  |
|  | 6 years to 9 years | 13 (18.3%) | 10 (20.8%) |  |
|  | More than 10 years | 22 (31.0%) | 16 (33.3%) |  |
| Working hours per day | Less than 6 hours | 2 (2.8%) | 1 (2.1%) | 0.977 |
|  | 6 to 9 hours | 52 (73.2%) | 34 (70.8%) |  |
|  | 10 to 12 hours | 12 (16.9%) | 9 (18.8%) |  |
|  | More than 12 hours | 5 (7.0%) | 4 (8.3%) |  |

Supplementary Table 2. Dietary patterns identified using Principal Component Analysis

| **Food groups** | **Western Pattern** | **Traditional Mixed Pattern** | **Protein-Rich Pattern** | **Fruit and Dairy Pattern** |
| --- | --- | --- | --- | --- |
| Sugary and Processed foods | .753 | .083 | .159 | -.220 |
| Beverages | .706 | .225 | -.127 | .431 |
| Dairy products | .669 | .267 | .092 | .256 |
| Alcoholic beverage | .550 | -.107 | .089 | -.050 |
| Animal proteins | .048 | .777 | .198 | .025 |
| Cereals and Grains | -.002 | .668 | -.256 | -.137 |
| Fats and oils | .205 | .634 | .371 | -.007 |
| Vegetables | -.057 | .608 | .371 | .396 |
| Tubers and Roots | -.048 | .148 | .689 | .142 |
| Legumes | .291 | .147 | .658 | .005 |
| Snacks and Street foods | .552 | -.160 | .556 | -.048 |
| Fruits | .009 | -.072 | .110 | .889 |
| % of variance | 26.733 | 14.352 | 10.125 | 9.173 |
| Cumulative % | 26.733 | 41.085 | 51.210 | 60.383 |

Supplementary Table 3. Multiple binary logistic associations between lifestyle factors, liver enzymes, and MetS

| **Lifestyle factors** | **MetS** | **ALT** | **AST** | **GGT** |
| --- | --- | --- | --- | --- |
| Work stress | 0.888 (0.157-5.020) 0.893 | 0.867 (0.081-9.329) 0.906 | 0.897 (0.039-20.568) 0.946 | 1.309 (0.250-6.867) 0.750 |
| Physical Activity | 1.000 (0.999-1.000) 0.308 | 1.000 (1.000-1.001) 0.428 | 1.000 (1.000-1.000) 0.373 | 1.000 (1.000-1.000) 0.620 |
| Western | 0.728 (0.384-1.380) 0.331 | 0.778 (0.339-1.787) 0.554 | 1.246 (0.455-3.410) 0.669 | 0.794 (0.424-1.485) 0.470 |
| Traditional mixed | 0.698 (0.351-1.386) 0.304 | 0.545 (0.199-1.490) 0.237 | 0.412 (0.105-1.609) 0.202 | 0.930 (0.482-1.793) 0.829 |
| Protein-rich | 1.247 (0.624-2.490) 0.532 | 0.542 (0.215-1.370) 0.195 | 1.299 (0.371-4.553) 0.683 | 1.239 (0.634-2.423) 0.531 |
| Fruit and Dairy | 1.440 (0.795-2.611) 0.229 | 1.442 (0.663-3.136) 0.355 | 1.114 (0.394-3.144) 0.839 | 1.174 (0.641-2.151) 0.603 |
